# Utilization and Factors Associated With Early Postnatal Care Services in Postpartum Mothers: A Cross-Sectional Study From Masafu General Hospital in Eastern Uganda

**DOI:** 10.7759/cureus.97463

**Published:** 2025-11-21

**Authors:** Caroline Natocho, Wycliffe S Wafula, Margaret Alioru, Vincent Mubangizi

**Affiliations:** 1 Faculty of Nursing and Midwifery, Clarke International University, Kampala, UGA; 2 Community Practice and Family Medicine, Mbarara University of Science and Technology, Mbarara, UGA

**Keywords:** institutional factors, maternal, postnatal care, postpartum, uganda

## Abstract

Background

Worldwide, postnatal care (PNC) has been declared to be the hallmark of mother-child health care after delivery. This study aimed to assess the utilization of early postnatal care (EPNC) services and identify the factors influencing their use among postpartum mothers at Masafu General Hospital in Busia District, Eastern Uganda.

Methodology

A cross-sectional, hospital-based study in a rural setting in Eastern Uganda was conducted in January 2025. A questionnaire was used to collect data from a sample of 234 postpartum women. Bivariate and multivariate logistic regressions were used to estimate factors associated with utilization of EPNC services, and variables with a p-value <0.05 were considered statistically significant.

Results

While 64.5% of postpartum mothers utilized EPNC services within the first week post-delivery, only 30.5% received care consistent with WHO recommendations for EPNC. Results showed that being aged between 18 and 27 and/or above 37 years (adjusted odds ratio (AOR) = 10.4, 95% confidence interval (CI) = 5.1-21.7, p = 0.01 and AOR = 18.5, 95% CI = 1.2-27.7, p = 0.04, respectively), mode of delivery (AOR = 16.0, 95% CI = 6.4-39.7, p = 0.00), maternal level of knowledge on EPNC (AOR = 5.8, 95% CI = 2.4-14.2, p = 0.01), long waiting time before receiving PNC services (AOR = 13.4, 95% CI = 1.9-9.6, p = 0.01), walking for less than 5 km to the health unit (AOR = 0.03, 95% CI = 0.0-0.6, p = 0.02), attending antenatal care (ANC) clinic at least four times (AOR = 0.01, 95% CI = 0.0-0.4, p = 0.01), attaining at more than primary level of education (AOR = 0.02, 95% CI = 0.0-0.6, p = 0.02), receiving PNC information from health workers during ANC/childbirth (AOR = 0.002, 95% CI = 0.0-0.0, p < 0.001), and being advised at discharge to turn up PNC follow-up (AOR = 0.1, 95% CI = 0.0-0.8, p = 0.03) were associated with increased odds of using EPNC.

Conclusions

Only about one-third of postpartum mothers utilized EPNC services three times, as recommended by the WHO. Key factors influencing EPNC were highlighted.

## Introduction

Worldwide, postnatal care (PNC) has been declared to be the hallmark of mother-child health care after delivery. Early postnatal care (EPNC) is the medical attention provided to the mother and newborn baby, particularly within the first seven days following childbirth [1]. The provision of EPNC is critical, as it aids in the early detection of post-delivery danger signs and management of post-delivery complications, which is necessary for promoting maternal-child health and preventing post-delivery deaths [2,3].

Globally, only about 30% of mothers follow recommended PNC, and the rate is lower (13%) in sub-Saharan Africa [4]. In Africa, there is low uptake of EPNC, reportedly ranging from 10% to 60%, which has been blamed on the low quality of services offered [5]. In Uganda and South Sudan, it was reported that only 15.4% and 11.4% of mothers got the required PNC within six days postpartum, respectively [6]. Inadequate EPNC utilization is a significant predictor of delayed detection of postpartum complications and an elevated risk of adverse maternal and newborn health outcomes. These include conditions such as puerperal sepsis, postpartum hemorrhage, and suboptimal breastfeeding practices, which collectively have detrimental effects on infant health and overall development. Such complications contribute a bigger share to mother-child morbidity and mortality during the postpartum period [7].

Regardless of the different measures that have been put on the ground, such as client health education during antenatal care (ANC) visits and mass media sensitization campaigns through various health programs like “obulamu,” the problem of low EPNC utilization among postpartum mothers occurs in various parts of Uganda, including at Masafu General Hospital. To bridge this identified gap, a study was undertaken to ascertain the utilization of EPNC services and the determinants influencing their uptake among postpartum mothers attending Masafu General Hospital, Busia District, Eastern Uganda. Data from the hospital’s HMIS report from April to June 2024 showed that only 48.2% (247) of mothers delivering at the facility during that period accessed EPNC. The substantial number of deliveries at this site, combined with the observed low EPNC utilization, establishes its strong suitability for this study’s sample recruitment.

The specific objectives of the study were to determine the proportion of postpartum mothers who utilize EPNC services among postpartum mothers attending Masafu General Hospital, Busia District; identify the sociodemographic factors influencing the utilization of EPNC services among postpartum mothers attending Masafu General Hospital, Busia District; and determine the institutional factors influencing the utilization of EPNC services among postpartum mothers attending Masafu General Hospital, Busia District.

## Materials and methods

Study design

This quantitative, cross-sectional study was conducted at a hospital in Eastern Uganda during January 2025.

Study setting

The study site was Masafu General Hospital, located in the rural Busia district, Eastern Uganda. Masafu General Hospital was established in 2010. The hospital has a 100-bed capacity. While Masafu General Hospital records an average of 18 daily deliveries, a critical challenge exists concerning postpartum mothers’ return for PNC, particularly EPNC. PNC follow-up is offered in a separate room. PNC services are provided at two hospitals, one health center IV, and eleven health centers IIIs in the district. The other hospital is a private Catholic mission hospital. However, Masafu serves the largest number compared to others.

Sample size determination

Postpartum mothers who delivered at Masafu General Hospital constituted the study population, as they were the direct beneficiaries and primary users of EPNC services. A sample size of 384 mothers was derived utilizing the Kish formula [8]: n = Z²pq/d². We used a Z score of 1.96 at a 95% confidence interval (CI). As the rate of EPNC uptake at Masafu General Hospital was not known, we used 50% to get the largest sample size. With the hospital’s reported average of roughly 20 daily deliveries, the targeted sample was attainable within a 30-day data collection window. The expected population of postpartum mothers was 600. Slovin’s formula was used [9]: nf = n/(1+n/N), where N is the accessible population (600), n is the determined sample size, 384, and nf is the sample size for the study. Therefore, a sample size of 234 postpartum mothers was considered for the study. The sample size was not calculated using the associated factors for utilization of EPNC.

Inclusion and exclusion criteria

The study included postpartum mothers attending Masafu General Hospital who consented to participate in the study. These mothers were attending a postnatal clinic for services such as general checks, mental health, nutrition support with micronutrient supplementation, family planning, and support and advice on breastfeeding. Further, mothers who brought their babies for the services, such as routine vaccination, growth and temperature monitoring, provision of tetracycline ointment and vitamin K, and monitoring and treatment of infection, were included. Mothers were excluded if they were acutely ill, if their child(ren) were critically ill, or if they had a cognitive impairment that precluded informed consent or interview participation.

Sampling technique

The study employed a systematic random sampling technique to recruit study participants, where respondents (postnatal mothers) who were attending Masafu General Hospital, Busia District, were selected.

Sampling procedure

For selection purposes, each mother was daily offered a box containing 30 slips, with 15 distinctly numbered and the remainder unnumbered. Mothers were briefed about the study and requested to select at random a piece of paper from a box without replacement. Those who picked numbered slips were considered for the study, and the numbers they picked were used as their identification numbers. This procedure was repeated until the predetermined sample size for the study was achieved.

Data collection tools

Data were collected from respondents using an interviewer-administered questionnaire. This instrument was specifically developed for the study, drawing upon existing literature to assess the level and associated factors of EPNC service utilization among postpartum mothers. Variables were dichotomously assessed as either present (yes) or absent (no).

The dependent variable was the utilization of EPNC services among post-delivery mothers attending Masafu General Hospital. The independent variables with their definitions are described in the following section. Participants’ ages were self-reported in completed years. Marital status was categorized as married, never married, separated, and widowed. Formal education was defined as the number of years spent in school: none, 1-7 years (primary school), 8-11 years (secondary school), and 12 or more years (tertiary education). Employment status was categorized as unemployed and employed (we were interested in knowing if the person was receiving a regular payment). The place where the participant lived was classified as rural or urban. Information was collected on the number of living children (as mothers could lose children even after the neonatal period), the number of times the mother attended ANC during the pregnancy, the type of delivery (classified as normal vaginal delivery or through operation), and whether the person was required to pay for EPNC.

Distance from the place of residence to the nearest health facility was ascertained by asking participants to estimate the distance as less than 1 km, 2-4 km, or 5 km and above. The place of delivery was assessed by inquiring whether the birth occurred at home or at a health facility. Participants were asked to report their waiting time for PNC services, which was categorized as less than 30 minutes, 30-60 minutes, or exceeding 60 minutes. To assess potential influencing factors, the study gathered information on mothers’ engagement with health worker-provided PNC support mechanisms. Participants were asked if they received PNC-specific information from health workers, were counseled to return for a follow-up/PNC appointment, received telephone reminders for PNC visits, benefited from health workers’ home visits for PNC, and identified health worker shortage at the hospital as a barrier to PNC access.

We asked mothers if they accessed the health information through media such as radio and television. We assessed participants’ perception of the importance of attending PNC and their willingness to recommend peers to attend all PNC visits. Responses were captured using a five-point Likert scale: strongly agree, agree, neutral, disagree, and strongly disagree [10]. The Likert scale is free to use, and there is no need for a licence. Mothers’ knowledge of EPNC was assessed by inquiring about the definition of EPNC (medical care from birth up to one week postpartum), the recommended number of PNC visits, the benefits of EPNC for the mother, and complications associated with inadequate PNC attendance. Scores were calculated as percentages and subsequently graded into three levels: low (<50%), moderate (50-70%), and high (>70%). The proportion of mothers using EPNC was determined by asking whether they received a medical check within seven days after delivery; if yes, respondents were asked how many checks occurred. Participants were categorized by socioeconomic status based on their self-reported daily expenditure, aligning with the World Bank’s international poverty lines [11]. Categories included were extremely poor (less than $1.90 (approximately Uganda shillings (UGX) 7,000) per day), poor ($1.90-$3.00 (approximately UGX 7,000-11,000) per day), and rich (more than $3.00 (approximately UGX 11,000) per day).

The study tool was administered in English or in the local language (Lusamia). The survey questionnaire was initially developed in English, then translated into Lusamia, and subsequently back-translated to English to ensure fidelity to the original wording.

Data collection procedure

Respondents were identified at the facility with assistance from the PNC in charge, who introduced the research team to the unit. Data were collected by two female interviewers with a diploma in midwifery. They were trained in the principles of research conduct and how to use the designed research tool. The questionnaire was interviewer-administered, with questions read aloud to each respondent in their preferred language. Responses were collected and recorded with precision.

Quality control

The validity of the study instrument was evaluated through two key processes: expert reviews by 10 experienced health workers and pilot testing. Pretesting of the data collection tools was conducted at a different general hospital, involving approximately 10% of the estimated sample size, to identify and resolve any ambiguities before the main study.

Data management and analysis

Data were cleaned and entered in Epi Data V.3.1. Data were exported and analyzed using SPSS version 26 (IBM Corp., Armonk, NY, USA). The first level of analysis was univariate analysis, where descriptive statistics were used to present results. Bivariate and multivariate analyses were performed to assess the association between the independent factors and EPNC variables, with a p-value less than 0.05 in bivariate analyses proceeding to multivariate modeling. Adjusted odds ratios (AORs) and their corresponding 95% CIs are reported.

Ethical considerations

Ethical approval to conduct this study was obtained from the Clarke International University Research and Ethics Committee (reference number: Clarke 2024-11). This study was conducted in accordance with the principles of the Declaration of Helsinki. We obtained administrative permission from the hospital medical superintendent. Study participants provided written informed consent to participate. If a signature could not be obtained for literacy reasons, verbal informed consent was obtained in the presence of a witness, and the study participant indicated a signature using a thumbprint.

## Results

Demographic characteristics of the study sample

The study enrolled 234 respondents (Table 1), with all approached potential participants agreeing to be interviewed. About 37.6% of respondents were aged 18-27 years, and the majority (80.8%) were married. A substantial proportion (52.6%) were unemployed. Only 0.9% of the participants had no formal education, while 55.1%, 23.9%, and 20.1% had attained primary, secondary, and tertiary education, respectively. About 82.1% of participants resided in rural areas.

**Table 1:** Sociodemographic characteristics of study participants (N = 234).

Variable	Frequency (n = 234)
Age group	
15–18 years	27 (11.5)
18–27 years	88 (37.6)
28–37 years	84 (35.9)
38 years and above	35 (15.0)
Level of education	
No formal education	2 (0.9)
Primary	129 (55.1)
Secondary	56 (23.9)
Tertiary	47 (20.1)
Type of employment	
Unemployed	123 (52.6)
Employed	121 (47.4)
Marital status	
Married	189 (80.8)
Never married	31 (13.2)
Separated	9 (3.8)
Widowed	5 (2.1)
Area of residence	
Rural	192 (82.1)
Urban	42 (17.9)

Proportion of postpartum mothers who utilized early postnatal care services

Only 64.5% of the participants accessed EPNC services within the first postpartum week. Among those who received EPNC, 53 (35.1%), 52 (34.4%), and 46 (30.5%) attended once, twice, and thrice, respectively.

Factors linked with utilization of early postnatal care services

The factors associated with utilization of EPNC among postpartum mothers at the bivariate and multivariate levels are shown in Table 2. Bivariate analysis showed a significant association between EPNC utilization and age, education, maternal attitudes and perceptions regarding PNC, and antenatal and delivery care history (number of ANC visits, type and place of delivery, and PNC information received during ANC/childbirth). Furthermore, EPNC access-related factors were significant, including maternal knowledge about EPNC, payment for healthcare services (such as EPNC), distance to the health unit, waiting time for PNC services, healthcare worker advice for return PNC visits at discharge, and receipt of post-childbirth home visits from health workers.

**Table 2:** Bivariate and multivariate analysis of factors associated with EPNC utilization. ANC = antenatal care clinic; COR = crude odds ratio; AOR = adjusted odds ratio; CI = confidence interval; EPNC = early postnatal care services; HCWs = healthcare workers; PNC = postnatal care services

Variable	Mother received EPNC		COR (95% CI)	P-value	AOR (95% CI)	P-value
	Yes, n (%)	No, n (%)				
Age group						
15–18 years	9 (3.9)	18 (7.7)	1	-	1	-
18–27 years	64 (27.4)	24 (10.3)	5.3 (2.1-13.5)	0.00	10.4 (5.1-21.7)	0.01
28–37 years	47 (20.1)	37 (15.8)	2.5 (1.0-6.3)	0.04	10.9 (0.4-27.4)	0.15
38 years and above	31 (13.3)	4 (1.7)	15.5 (4.2-57.6)	<0.0001	18.5 (1.2-27.7)	0.04
Level of education						
None	0 (0.0)	2 (0.9)	1	-	1	-
Primary	67 (28.6)	62 (26.5)	3.2 (0.3-31.9)	0.31	0.01 (0.0-1.2)	1.00
Secondary	45 (19.2)	11 (4.7)	11.5 (1.1-120.7)	0.04	0.02 (0.0-0.6)	0.02
Tertiary	39 (16.7)	8 (3.4)	13.3 (1.2-143.5)	0.03	0.01 (0.0-0.3)	0.01
Employment status						
Unemployed	73 (31.2)	50 (21.3)	1	-	-	-
Employed	78 (33.3)	33 (14.1)	1.0 (0.6-1.6)	0.98	-	-
Marital status						
Married	123 (52.6)	66 (28.2)	1	-	-	-
Never married	19 (8.1)	12 (5.1)	1.2 (0.5-2.6)	0.68	-	-
Separated	6 (2.6)	3 (1.3)	0.9 (0.2-3.9)	0.92	-	-
Widowed	3 (1.3)	2 (0.9)	1.2 (0.2-7.6)	0.82	-	-
Residence						
Rural	122 (52.1)	70 (29.9)	1	-	-	-
Urban	29 (12.4)	13 (5.6)	0.8 (0.4-1.6)	0.50	-	-
Number of living children						
1	25 (10.7)	21 (9.0)	1	-	-	-
2–4	95 (40.6)	44 (18.8)	0.6 (0.3-1.1)	0.09	-	-
5 or more	31 (13.3)	18 (7.7)	1.5 (0.6-3.3)	0.38	-	-
Having access to health information via the media						
Yes	118 (50.4)	70 (29.9)	1	-	-	-
No	33 (14.1)	13 (5.6)	0.7 (0.3-1.4)	0.26	-	-
Attending PNC is perceived as important, and the willingness to recommend to peers						
Strongly agrees	81 (34.6)	18 (7.7)	1	-	1	-
Agrees	50 (21.4)	43 (18.4)	0.3 (0.1-0.5)	<0.0001	5.0 (0.21.2)	0.16
Neutral	16 (6.8)	13 (5.6)	0.3 (0.1-0.7)	0.00	11.7 (0.6-2.5)	0.08
Disagrees	0 (0.0)	7 (3.0)	0.0 (0.0-0.2)	0.00	4.1 (1.0-1.7)	0.06
Strongly disagrees	4 (1.7)	2 (0.9)	0.4 (0.1-2.6)	0.37	8.5 (0.0-3.1)	1.00
Number of ANC visits attended						
Once	4 (1.7)	3 (1.3)	1	-	1	-
Two to three visits	33 (14.1)	8 (3.4)	3.1 (0.6-16.7)	0.19	0.01 (0.0-0.1)	0.59
Four or more visits	114 (48.7)	72 (30.8)	1.2 (2.5-5.3)	0.05	0.01 (0.0-0.4)	0.01
Type of delivery						
Normal vaginal delivery	105 (44.9)	77 (32.9)	1	-	1	-
Caesarean section	46 (19.7)	6 (2.6)	0.2 (0.1-0.4)	0.00	16.0 (6.4-39.7)	0.00
Level of knowledge about EPNC						
Low (<50%)	20 (8.6)	36 (15.4)	1	-	1	-
Moderate (50–70%)	51 (21.8)	32 (13.7)	2.9 (1.4-5.8)	0.00	5.8 (2.4-14.2)	0.01
High (>70%)	80 (34.2)	15 (6.4)	9.6 (4.4-20.9)	<0.0001	4.2 (0.8-22.2)	0.09
Paid for services like EPNC						
Yes	25 (10.7)	6 (2.6)	1	-	1	-
No	126 (53.9)	77 (32.9)	2.6 (1.0-6.5)	0.04	0.8 (0.1-10.8)	0.83
Wealth status of the mother						
Extremely poor	23 (9.8)	13 (5.6)	1	-	-	-
Poor	102 (43.6)	60 (25.6)	1.0 (0.5-2.0)	0.92	-	-
Rich	26 (11.1)	10 (4.3)	1.5 (0.5-4.0)	0.45	-	-
Got support from the husband or relatives to seek EPNC						
Yes	127 (54.3)	64 (27.4)	1	-	-	-
No	24 (10.3)	19 (8.1)	1.6 (0.8-3.1)	0.19	-	-
Distance travelled to the hospital						
≤1 km	30 (12.8)	7 (3.0)	1	-	1	-
2–4 km	43 (18.4)	35 (15.0)	0.3 (0.1-0.7)	0.01	0.03 (0.0-0.6)	0.02
5 km and more	78 (33.3)	41 (17.5)	0.4 (0.2-1.1)	0.08	0.6 (0.0-9.4)	0.71
Place of delivery						
Home	6 (2.6)	4 (1.7)	1	-	1	-
Health facility	145 (62.0)	79 (33.7)	0.8 (0.2-1.0)	0.04	0.0 (0.2-1.0)	1.00
Received information on PNC from the health workers during ANC and childbirth						
Yes	117 (50.0)	17 (7.3)	1	-	1	-
No	34 (14.5)	66 (28.2)	13.4 (7.0-25.7)	<0.0001	0.0 (0.0-0.0)	<0.001
Waiting time to get PNC						
Less than 30 minutes	72 (30.8)	7 (3.0)	1	-	1	-
30 minutes to 1 hour	47 (20.1)	65 (27.8)	0.1 (0.0-0.2)	<0.0001	1.3 (0.1-2.0)	0.84
More than 1 hour	32 (13.7)	11 (4.7)	0.3 (0.1-0.8)	0.02	13.4 (1.9-9.6)	0.01
Advised to return for PNC by the HCWs on discharge						
Yes	118 (50.4)	27 (11.5)	1	-	1	-
No	33 (14.1)	56 (23.9)	7.4 (4.1-13.5)	<0.0001	0.1 (0.0-0.8)	0.03
Received reminder calls to return for a checkup						
Yes	26 (11.1)	9 (3.9)	1	-	-	-
No	125 (53.4)	74 (31.6)	1.7 (0.8-3.8)	0.19	-	-
Received a home visit from HCWs						
Yes	46 (19.7)	12 (5.1)	1	-	1	-
No	105 (44.9)	71 (30.3)	2.6 (1.3-5.2)	0.01	12.0 (0.8-17.5)	0.07
Failed to get PNC due to a few HCWs at this hospital						
Yes	54 (23.1)	38 (16.2)	1	-	-	-
No	97 (41.5)	45 (19.2)	0.7 (0.4-1.1)	0.13	-	-

The variables that were significant in the bivariate analysis were entered into a multivariate logistic regression model, and each variable remained significantly associated with EPNC utilization, except for having a positive attitude and good perception toward PNC and willingness to recommend peers to attend the same, payment for healthcare services (including EPNC), place of delivery, and receipt of home visits from health workers following the current childbirth.

Being aged between 18 and 27 and/or above 37 years (AOR = 10.4, 95% CI = 5.1-21.7, p = 0.01 and AOR = 18.5, 95% CI = 1.2-27.7, p = 0.04 respectively), mode of delivery (AOR = 16.0, 95% CI = 6.4-39.7, p = 0.00), maternal level of knowledge on EPNC (AOR = 5.8, 95% CI = 2.4-14.2, p = 0.01), long waiting time before receiving PNC services (AOR = 13.4, 95% CI = 1.9-9.6, p = 0.01), walking for less than 5 km to the health unit (AOR = 0.03, 95% CI = 0.0-0.6, p = 0.02), attending ANC clinic at least four times (AOR = 0.01, 95% CI = 0.0-0.4, p = 0.01 value), attaining more than primary level of education (AOR = 0.02, 95% CI = 0.0-0.6, p = 0.02), receiving PNC information from health workers during ANC/childbirth (AOR = 0.002, 95% CI = 0.0-0.0, p < 0.001), and being advised at discharge to turn up for PNC follow-up (AOR = 0.1, 95% CI = 0.0-0.8, p = 0.03) were associated with increased odds of using EPNC.

## Discussion

In this hospital-based study of 234 postpartum women, it was found that, overall, 64.5% of the respondents utilized EPNC services at one point during the first week of post-delivery. The findings revealed that only 30.5% of the postpartum mothers received the recommended EPNC in the first seven days of childbirth, which, according to WHO, was in agreement with other studies in sub-Saharan Africa [12]. The utilization rate was low compared to the 45.5% reported in a study from Ethiopia [13]. The possible reason for this gap could be differences in the sociodemographic characteristics of the study participants and the population across the countries.

The study further identified several independent factors associated with the utilization of EPNC. Mothers aged between 18 and 27 years were more likely to receive EPNC compared to other age groups. It is plausible that the elevated EPNC utilization among mothers in the 18-27-year age group is associated with their lower parity (often first or second deliveries). Such mothers may have limited experience with postpartum complications, thus actively seeking EPNC. Similarly, mothers aged 38 years and older were more likely to utilize EPNC. This trend might be explained by their higher parity (multiparous or grand multiparous), which could lead to greater accumulated knowledge about, and appreciation for, the full scope of EPNC benefits after delivery. Other studies have reported similar findings [14-17].

Maternal educational attainment was observed to have a statistically significant influence on EPNC utilization, a finding that corroborates existing literature. This is in line with findings from other studies [13,18-20]. This is likely because mothers with higher educational attainment are better equipped to access and comprehend health information, emphasizing the importance of attending EPNC. Conversely, mothers with no formal education may face significant barriers, particularly as much of this vital health information, including official communications and educational materials, is often produced primarily in English, thus limiting their access to crucial knowledge.

The study further found that the number of ANC visits the mother attended affected the utilization of EPNC services among post-delivery mothers. This study’s findings are in line with findings of other studies from African countries [21-24]. This effect is most likely because during ANC visits, pregnant mothers are given health education about a wide range of health issues, which include the need to attend PNC at birth and subsequent visits. The social environment of ANC visits likely contributes to enhanced awareness of PNC. These settings provide platforms for pregnant mothers to engage with peers, exchange insights on previous pregnancies and deliveries, and, consequently, develop a clearer understanding of their postpartum care needs.

Utilization of EPNC was associated with the type of delivery a mother had. Other studies from Uganda, Ethiopia, Tanzania, and Sierra Leone have found a similar finding [4,5,18,25]. The prolonged hospital stays for mothers recovering from the cesarean section offer an inherent opportunity for the provision of EPNC services, as these are typically delivered alongside their postoperative treatment. It is opined that mothers who deliver by cesarean section are given more instructions on discharge, among which include the need to return for more reviews than those who deliver normally.

Mothers’ knowledge of EPNC was associated with EPNC utilization, consistent with other studies [5,21,23,26]. It is plausible that higher maternal knowledge facilitates the accurate understanding of crucial EPNC messages, thereby enhancing awareness of the benefits for both postpartum well-being and newborn health outcomes. The study also showed that distance to the health facility affected EPNC utilization. This finding concurs with findings of other studies from sub-Saharan Africa [12,18,21,27]. Long distances to the health facility make it difficult for postnatal mothers to walk or travel.

A significant association was observed between mothers’ exposure to PNC information from health workers during ANC and the peripartum period and their subsequent utilization of EPNC. This increased likelihood may be attributed to the awareness and understanding of the importance of EPNC, which can significantly improve maternal and infant health outcomes. Therefore, enhancing outreach and education efforts during ANC visits is crucial for promoting EPNC utilization among new mothers. This finding is in support of earlier studies reported from Africa [13,25,28]. Educating mothers on PNC equips them with the knowledge that helps them make informed decisions regarding EPNC attendance. It also makes them aware of the likely dangers that can occur during postpartum, which necessitate them seeking PNC checkups.

Mothers who waited longer before receiving health care were more likely to use EPNC. This finding is counterintuitive because longer waiting times would normally be expected to reduce EPNC utilization, not increase it. Studies from Kenya and other parts of Uganda showed that long waiting times at the health units hindered EPNC attendance [29,30]. Perhaps mothers who cannot wait for longer hours at this rural hospital march away without receiving EPNC services, and this long waiting also discourages them from returning for subsequent checkups, especially when they have no serious health challenges.

The study further established that mothers who were not advised to return for PNC checkups by the health workers on discharge were less likely to attend EPNC. This was in support of a study from Ethiopia [13]. It can be opined that when postpartum mothers are advised to return for checkups and appointment dates are given, it emphasizes the need to attend EPNC.

Study strengths and limitations

The study’s strengths include the use of brief patient‑reported measures that are practical for busy primary care settings. Recruiting the sample from clinics enhanced sample diversity. The analysis linking patient‑reported well‑being to health outcomes in an understudied population.

Several limitations affect the interpretation of our findings. The study variables were self-reported. Asking participants about past behaviors may have introduced recall bias. To mitigate this, we encouraged participants to reference personal documentation, ensuring the information was within the past year. Social desirability bias may have affected the study findings. To address this, we ensured anonymity and confidentiality during data collection to make respondents feel more comfortable being honest.

The study was done only in one hospital, and thus, the findings may not apply beyond the specific context of Masafu General Hospital and Busia District, especially for urban settings or other parts of Uganda. The findings may not apply to mothers who do not use formal healthcare facilities. Questions assessing complex concepts such as knowledge or perception may not fully capture the intended variables. To mitigate against this, we validated our study tools for measuring these variables and revised questions based on pretesting feedback. This study primarily gathered data from postpartum mothers, omitting the perspectives of healthcare providers involved in their care at the facility. We did not include variables such as medical disease, complications during pregnancy and labor, and previous pregnancy outcomes.

Despite these limitations, the insights gained into factors influencing EPNC utilization within a resource-constrained setting like Uganda remain critical. This understanding is fundamental for informing public awareness campaigns, guiding policy recommendations, and developing effective interventions. Researchers should conduct further studies on PNC utilization among mothers who do not visit formal healthcare facilities. Such studies would provide a broader understanding of barriers and facilitators outside hospital settings. It is advised that future studies examine the intricate interactions between regional customs, beliefs, and practices and how they affect PNC services’ perception and use. Furthermore, it would be valuable to assess the quality and adequacy of EPNC services currently available in rural hospitals, determining their role in influencing service uptake and continuity of care.

## Conclusions

This study revealed low utilization of EPNC services, with only 30.5% receiving care within the WHO-recommended first week. Key factors influencing EPNC attendance included maternal age, education level, ANC attendance, delivery type, knowledge level, and facility proximity. Barriers such as distance and insufficient sensitization highlight areas for intervention, making addressing these barriers critical. Further research on non-facility users and broader sociocultural dynamics can shape strategies to improve maternal and newborn health outcomes.
